# A Novel Variant of Avian Reovirus Is Pathogenic to Vaccinated Chickens

**DOI:** 10.3390/v15091800

**Published:** 2023-08-24

**Authors:** Rui Liu, Dan Luo, Jinhui Gao, Kai Li, Changjun Liu, Xiaole Qi, Hongyu Cui, Yanping Zhang, Suyan Wang, Xiaomei Wang, Yulong Gao, Li Gao

**Affiliations:** 1 Division of Avian Immunosuppressive Diseases, State Key Laboratory for Animal Disease Control and Prevention, Harbin Veterinary Research Institute, Chinese Academy of Agricultural Sciences, Harbin 150069, China; lrhhxxttxs@163.com (R.L.); ldfdan@163.com (D.L.); gao1241707546@163.com (J.G.); likai01@caas.cn (K.L.); liuchangjun@caas.cn (C.L.); qixiaole@caas.cn (X.Q.); cuihongyu@caas.cn (H.C.); zhangyanping03@caas.cn (Y.Z.); wangsuyan@caas.cn (S.W.); wangxiaomei@caas.cn (X.W.); 2 Jiangsu Co-Innovation Center for Prevention and Control of Important Animal Infectious Disease and Zoonoses, Yangzhou University, Yangzhou 225009, China

**Keywords:** avian reovirus, variant, pathogenicity, immunized chickens

## Abstract

Avian reovirus (ARV) infections, characterized by severe arthritis, tenosynovitis, pericarditis, and poor weight gain, have become increasingly serious in recent years. The economic impact is significant as it causes growth inhibition and immunosuppression. Some commercial poultry in China have been widely vaccinated with available ARV vaccines; however, infections continue to occur even after vaccination. This study aimed to isolate a novel variant, ARV-SD19/11103, from the joint tissues of infected broiler chickens vaccinated with ARV vaccines in Shandong Province. Genetic evolution analysis of the major protective antigen σC gene in ARVs showed that ARV-SD19/11103 was located in the genotype cluster I but not in the same sub-cluster as the S1133 vaccine strain. The amino acid sequence similarity between SD19/11103 and vaccine strains S1133, 1733, and 2408 was <80%. After analyzing the amino acid sequences of the σC protein, 33 amino acid differences were found between the new variant isolate and the vaccine strains. This novel variant showed obvious pathogenicity in specific pathogen-free chicken embryos and chicks and could cause serious disease in chickens vaccinated with commercially available ARV vaccines. Cross-neutralization experiments further demonstrated a significant antigenic difference between the novel variant and genotype cluster I ARV strains. The novel variant strain isolated in this study provides an important theoretical basis for understanding the prevalence and genetic evolutionary characteristics of ARV variant strains in our country. This study identified the causes of ARVs circulating and emphasizes the needs for developing new vaccines against novel ARV variants.

## 1. Introduction

Avian reovirus (ARV) is an RNA virus belonging to the genus *Orthoreovirus* and family *Reoviridae* [1]. Since Fahey and Crawley first isolated ARV in 1954 [2], outbreaks of ARV infections have been reported worldwide, posing a new challenge to the poultry industry [3,4,5,6,7]. ARV infection typically leads to a syndrome of growth retardation and immunosuppression in young chickens, with severe arthritis and tenosynovitis being the most common clinical symptoms, which have significant impact on the poultry industry. ARV infection induces immunosuppression in chickens, which leads to reduced immune function and increased susceptibility to other diseases.

ARV is a double-stranded RNA virus with a genome consisting of 10 segments. Based on the different migration rates of nucleic acid electrophoresis, the 10 segments were classified into three groups: L (large, including L1, L2, and L3), M (medium, including M1, M2, and M3), and S (small, including S1, S2, S3, and S4). The viral capsid protein σC, encoded by the S1 segment, is closely related to the infection, pathogenicity, and antigenic variation of ARV [8], which can induce the production of neutralizing antibodies in the body and play an important role in virus transmission and infection [1,6,9,10,11,12]. Virus isolation from the tendons/synovial fluid of clinically affected birds is the gold standard for diagnosis of reoviruses in clinical cases of viral arthritis/tenosynovitis. Following isolation of reovirus, genetic characterization, based on RT-PCR amplification of the σC protein followed by sequencing, is utilized to genotype isolates. According to σC genetic evolutionary analysis, ARVs can be classified into six genotype clusters, and current commercial vaccine strains belong to genotype cluster I (such as S1133, 1733, and 2408) [4,13,14].

As commercial ARV vaccines are becoming popular and biosecurity measures improve, many countries have successfully controlled the genotype cluster I ARV strains. Vaccination with both live attenuated and inactivated oil emulsion vaccines have been used successfully for decades to control the disease. Current commercial vaccine strains belong to the same serotype and are antigenically and serologically distinct from circulating variant field viruses isolated from clinical cases of tenosynovitis. Since 2012, there has been a dramatic increase in the number of clinical cases of tenosynovitis in commercial poultry and commercial vaccines are unable to provide adequate levels of protection against disease. However, the emergence of ARV variants has resulted in a continuously increasing incidence of ARV-induced diseases with diverse clinical manifestations, posing a serious threat to the poultry industry.

This study aimed to demonstrate the relationship between clinical cases of viral arthritis in China and the existence of ARV variants, and to investigate whether new ARV variants can evade immune protection conferred by commercial ARV vaccines.

## 2. Materials and Methods

### 2.1. Viruses and Vaccine

The ARV-HeB02 strain [7] was isolated and identified at the Division of Avian Immunosuppressive Diseases, Harbin Veterinary Research Institute, Chinese Academy of Agricultural Sciences. The immune protection assay was performed using commercial ARV-inactivated vaccine (S1133 strain).

### 2.2. Cells and Animals

Male Leghorn chicken hepatocellular carcinoma (LMH) cells (CRL-2117) were purchased from the American Type Culture Collection (ATCC) and preserved in our laboratory. Specific pathogen-free (SPF) chicken embryos and SPF chickens were purchased from the National Laboratory Poultry Animal Resource Center (Harbin, China). This study was conducted in accordance with the recommendations of the Guide for the Care and Use of Laboratory Animals of the Ministry of Science and Technology of China. The use of SPF chickens was approved by the Animal Ethics Committee of Harbin Veterinary Research Institute of the Chinese Academy of Agricultural Sciences and was performed in accordance with animal ethics guidelines and approved protocols (SYXK (Hei) 2017-009).

### 2.3. Clinical Samples and Virus Isolation

Ten clinical joint samples were collected from suspected cases of viral arthritis and tenosynovitis in broiler chickens from a vaccinated farm in Shandong province, China. Joint tissues were homogenized in phosphate-buffered saline (PBS) containing 10% (*v*/*v*) penicillin and streptomycin. The homogenate was frozen and thawed three times, centrifuged at 8000× *g* at 4 °C for 10 min, and the supernatant was harvested. The tissue supernatant was filtered, sterilized, and inoculated onto a 6-well plate with a monolayer of LMH cells at a ratio of 10% (*v*/*v*). The plates were continuously cultured at 37 °C and 5% CO_2_ for 5 days, and the growth and cytopathic effects of the cells were observed daily. When the cytopathic effects reached 80% or more, the cell culture was collected, blind passaged for three generations, and stored at −20 °C for subsequent use.

### 2.4. Immunofluorescence Assay

To confirm viral isolation and evaluate the antigenicity of the ARV isolate SD19/11103, an immunofluorescence assay (IFA) was performed. HeB02 was used as a positive control. LMH cells were infected with 1 × 10^4.0^ TCID_50_ of SD19/11103 or HeB02. Twenty-four hours later, cells were harvested and fixed with 4% paraformaldehyde for 30 min and permeabilized with 0.1% Triton X-100 in PBS for 15 min, followed by blocking with 5% bovine serum albumin in PBS for 1 h. Then, the cells were incubated with monoclonal antibody against ARV σB or ARV-positive sera diluted in PBS for 1 h. The cells were washed five times with PBS and incubated with the fluorescein isothiocyanate-conjugated anti-mouse or anti-chicken IgG (Sigma, St. Louis, MO, USA) secondary antibodies. Finally, the cells were washed five times with PBS and observed under a fluorescent inverted microscope (Thermo Fisher Scientific, Waltham, MA, USA).

### 2.5. Viral RNA Extraction, Reverse Transcription-Polymerase Chain Reaction (RT-PCR), and Sequencing

Total viral RNA was extracted from the culture supernatant using RNAiso Plus (Takara, Beijing, China). cDNA was synthesized from viral RNA using ReverTra Ace^®^ qPCR RT Master Mix and gDNA Remover (TOYOBO, Osaka, Japan). A pair of specific primers, GC1F (5′-ATGGCGGGTCTCAATCCATCGCAGCG-3′) and GC1R (5′-TTAGGTGTCGATGCCGGTACGCACGG-3′), designed based on the σC gene sequence of the ARV S1133 strain (L39002), were used for PCR amplification with the above cDNA as the template. The PCR was performed at 95 °C for 5 min followed by 35 cycles at 95 °C for 30 s, 55 °C for 30 s, and 72 °C for 1 min, with a final extension at 72 °C for 10 min. The amplified PCR fragments were cloned into the pMD18-T vector (Takara, China) and transformed into DH5α Escherichia coli cells, and the correct clones were sequenced by Comate Biosciences Company (Changchun, China).

### 2.6. Sequence Analysis

The nucleotide sequences of the σC gene of the isolated strain were analyzed for genetic evolution and homology using the DNAStar and MEGA programs (version 6.0) [15], and the confidence levels were assessed using 1000 bootstrap replicates [16]. The phylogenetic evolutionary trees were visualized using Interactive Tree of Life (iTOL) (https://itol.embl.de/ (accessed on 13 August 2023)). The reference strains used in this study and their GenBank accession numbers were listed in the Supplementary Table S1. 

To accurately describe the genomic characteristics of SD19/11103, 21 genotype cluster I ARV strains were selected for further analysis using DNAStar and MEGA program (version 6.0).

### 2.7. Pathogenicity Evaluation of the Isolated Strain

To evaluate the pathogenicity of this isolated variant, animal experiments were conducted using the variant strain SD19/11103 and the genotype cluster I strain HeB02. One-day-old SPF chicks were randomly divided into three groups, a blank control group and two ARV infection groups, with 15 chicks in each group. Each chicken of the infection group was inoculated with a dose of 1 × 10^6.0^ TCID_50_ (100 μL) SD19/11103 or HeB02, whereas the control group was inoculated with an equal volume of sterile PBS. Each chicken was inoculated on its left footpad, with the right footpad left uninoculated as a control. The chicks were observed for 14 days to calculate the mortality rate.

### 2.8. Cross-Neutralization Assay

To evaluate the antigenic differences between the novel ARV variant and genotype cluster I ARV, the antigenic relationships between SD19/11103 and HeB02 were analyzed via cross-neutralization assay using positive sera against each of the two virus strains. Titers of the SD19/11103 and HeB02 strains were determined in LMH cells and diluted to 200 TCID_50_/100 μL in Dulbecco’s Modified Eagle Medium containing 2% (*v*/*v*) fetal bovine serum and 1% (*v*/*v*) penicillin and streptomycin. All serum samples were subjected to two-fold serial dilutions in a blank 96-well plate. The diluted virus was mixed with sera and incubated for 1 h, and then added to a 96-well plate containing LMH cells. The cells were cultured for 5 days, cytopathic effects were observed, and the neutralizing titer was calculated. The experiments were replicated thrice with three samples of each.

### 2.9. Immune Protection Assay

To evaluate the protection of commercial ARV vaccine against this isolated variant, SPF chickens aged 3 weeks were randomly divided into five groups with 10 chickens in each group. Group 1 and group 2 were immunized with commercial vaccine at 3 weeks of age and received secondary immunization at 6 weeks of age following the use instructions of the vaccine. Groups 3 to 5 received an equal volume of PBS and served as control groups. At 9 weeks of age, group 1 and group 3 were challenged with 1 × 10^4.5^ ELD_50_ (100 μL) of SD19/11103, and group 2 and group 4 were challenged with 1 × 10^4.5^ ELD_50_ (100 μL) of HeB02. Group 3 and group 4 served as challenge controls. Group 5 received equal volume of PBS and served as a blank control. All the chickens were challenged on the left footpad and the right footpad was not challenged and was used as a control. All chickens were numbered and observed daily for 10 days for clinical signs and morbidity. At 5 days post-infection (5 dpi) and 10 dpi, three chickens were randomly selected from each group and euthanized for necropsy and pathological changes observation. GraphPad Prism software (version 8.0) was used to plot the morbidity rate curves and column charts of the commercial vaccine protection rates.

## 3. Results

### 3.1. SD19/11103 Isolation and Identification

**Figure 1:**
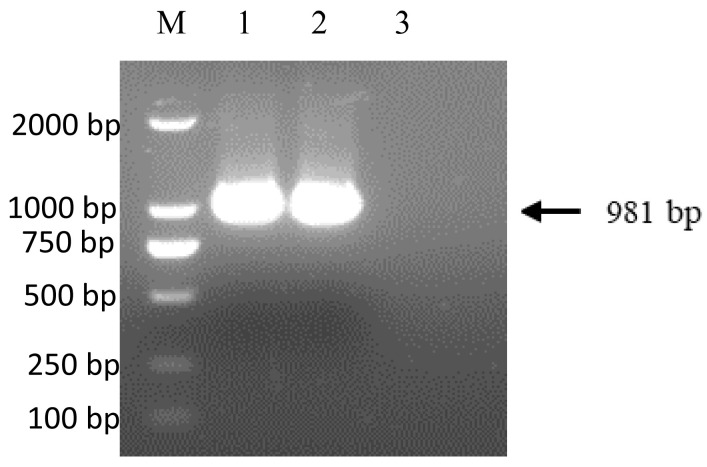
Amplification of σC gene using RT-PCR. A specific fragment of 981 bp was amplified from the SD19/11103 strain or positive control.

**Figure 2:**
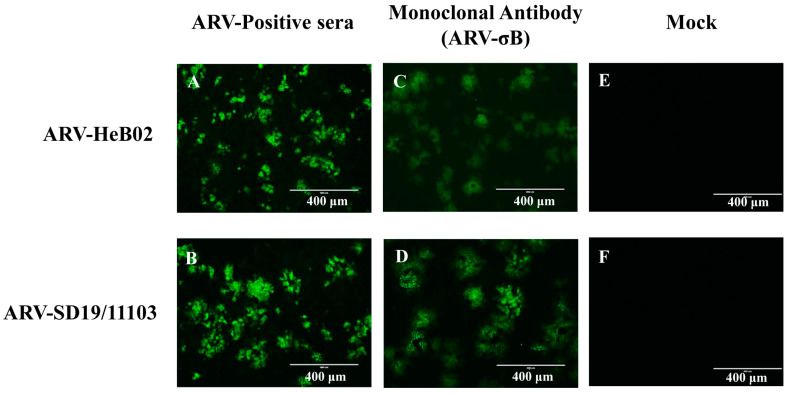
The results of immunofluorescence assay. LMH cells were infected with the ARV variant (SD19/11103) or the genotype cluster I ARV strain (HeB02). The monoclonal antibody against ARV σB protein or ARV-positive sera were used as the primary antibody, and fluorescein isothiocyanate-conjugated anti-mouse or anti-chicken IgG was used as the secondary antibody. The scale bars were 400 μm. (**A**–**D**) Positive green fluorescence signals were observed in ARV-infected cells. (**E**,**F**) No fluorescent signals were detected in control LMH cells.

A total of 10 clinical joint samples with suspected ARV infected were collected from a broiler enterprise in Shandong Province of China in 2019, which has been vaccinated with ARV commercial vaccines. The symptoms of the diseased broilers were mainly characterized by lameness and tarsal joint swelling/deformation. During the autopsy of the joints, a substantial quantity of purulent, blood-like mucus or gelatinous exudate was observed in the joint cavity. Joint tissues were homogenized in phosphate-buffered saline (PBS) and the supernatant was harvested and filtered. The tissue suspension was inoculated on LMH cells, which were collected when cytopathic effects appeared. The isolated virus was confirmed using RT-PCR and IFA. Specific bands of 981 bp were amplified from the culture cells and the positive control HeB02 (Figure 1), which was consistent with the expected size. No specific band was observed in the negative control. Green fluorescence signals were detected in LMH cells infected with both ARVs, whereas no green fluorescence signals were observed in the control group (Figure 2), further confirming the successful isolation of the ARV strain. The isolated strain was named ARV-SD19/11103.

### 3.2. SD19/11103 Evolutionarily Belongs to the Variant ARV

**Figure 3:**
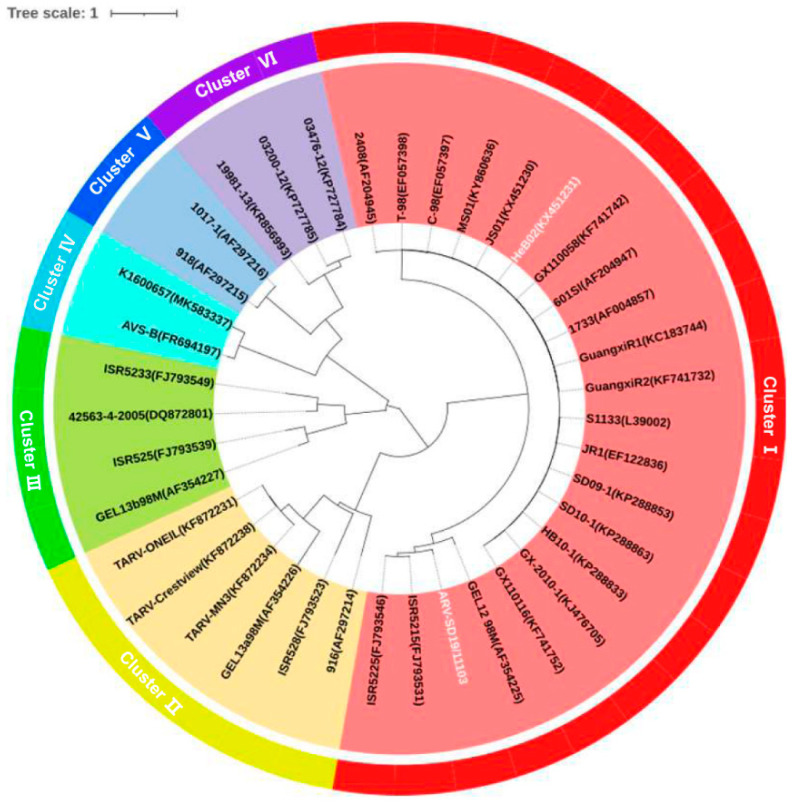
Phylogenetic analysis of nucleotide sequences of σC gene. The phylogenetic tree was generated using a neighbor-joining method with 1000 bootstrap replicates. The tree was visualized via iTOL (https://itol.embl.de/ (accessed on 13 August 2023).). The novel variant SD19/11103 and genotype cluster I strain HeB02 were highlighted in white.

The evolutionary relationship between SD19/11103 and the reference ARV strains was analyzed based on the nucleotide sequences of the σC gene. The neighbor-joining method was used to construct a phylogenetic tree. As shown in Figure 3, the ARV strains clustered into six genotypes. The SD19/11103 strain isolated in this study is located in the genotype cluster I, comprising vaccine strains S1133, 1733, and 2408, but not in the same sub-cluster as the vaccine strain S1133. The amino acid sequences similarity between SD19/11103 and the vaccine strains S1133, 1733, and 2408 was <80% (73.1–76.1%). The highest similarities (>90%) were detected between SD19/11103 and the variant strains ISR5225 and ISR5215 isolated in Israel (91.4–91.5%). 

To further study the molecular characteristics of SD19/11103, the amino acids of the σC gene were analyzed. There were 33 different amino acid sites between SD19/11103 and the genotype cluster I ARV strains (such as the vaccine strain S1133). These differences were located at 26, 27, 46, 49, 51, 54, 60, 68, 70, 77, 78, 83, 86, 94, 95, 98, 102, 111, 112, 126, 137, 147, 156, 203, 236, 243, 245, 256, 261, 264, 285, 291, and 310 (Supplementary Table S2).

### 3.3. SD19/11103 Is Pathogenic to SPF Chickens

**Figure 4:**
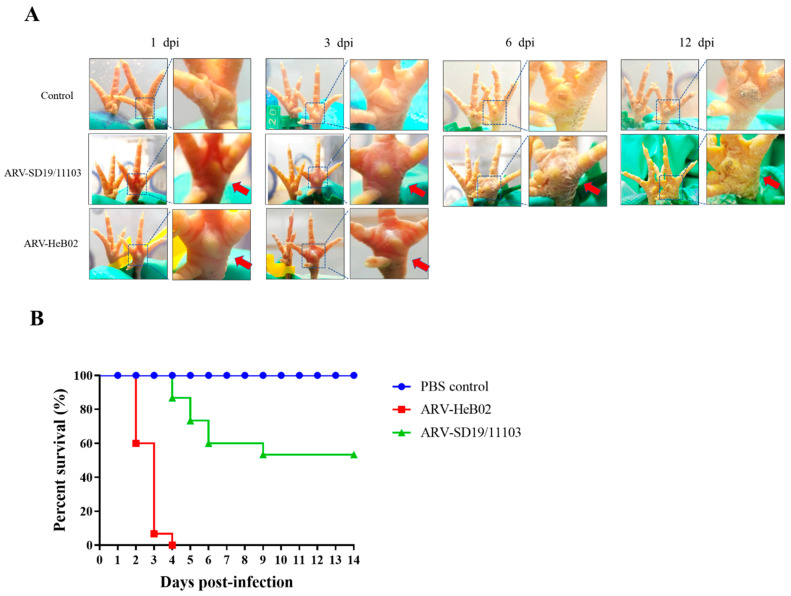
Evaluation of the pathogenicity of the variant strain SD19/11103 in one-day-old SPF chicks. (**A**) The gross lesions of paw pads of different groups were observed at different time points after infection with the SD19/11103 or HeB02 strain. All infected chickens showed clinical symptoms such as depression and severe swelling of the footpads during the course of the experiment. Infected chicks showed the most severe footpad symptoms at 4–6 days after infection, with red and swollen feet. The footpad symptoms gradually improved and showed slight swelling from 10 days after infection. Chicks infected with the SD19/11103 strain exhibited a slightly delayed time of death compared to those infected with the HeB02 strain. To clearly show the reddish and swelling foot, parts of the images have been enlarged and indicated with red arrows. (**B**) Percent survival of SPF chickens after infection with the SD19/11103 or HeB02 strain.

To investigate the pathogenicity of SD19/11103 in SPF chickens, the genotype cluster I strain HeB02 was used as a control. All infected chickens showed clinical symptoms such as depression and severe swelling of the footpads during the course of the experiment. Infected chicks showed the most severe footpad symptoms at 4–6 days after infection, with red and swollen feet. From 10 days after infection to the end of the observation period, the footpad symptoms gradually improved and showed slight swelling (Figure 4A). Chicks infected with the SD19/11103 strain exhibited a slightly delayed time of death compared to those infected with the HeB02 strain. The mortality rate of SPF chickens infected with SD19/11103 was 46.7%. Peak mortality occurred on the fifth day after infection, and no deaths occurred after 9 days. All chicks in the HeB02 strain-infected group died within 4 days after infection (Figure 4B), with a mortality rate of 100%. Throughout the experimental period, the chicks in the control group showed no signs of illness or death (Figure 4). The result showed that the variant strain SD19/11103 was pathogenic to SPF chickens.

### 3.4. SD19/11103 Showed Obvious Antigenicity Different Compared with Genotype Cluster I ARV

To detect antigenic differences between the novel variant ARV and the genotype cluster I ARV strain, the antigenic relationship between SD19/11103 and HeB02 was analyzed using a cross-neutralization test. To assess the potential serological cross-neutralization between SD19/11103 and HeB02, neutralization assays were performed using serum samples from SD19/11103-infected and HeB02-infected SPF chickens. The results revealed a weak cross-reactivity between SD19/11103 and HeB02. The neutralizing antibody titer of SD19/11103 sera against its self-virus was 2^6^, but the sera had no neutralizing ability against HeB02. The neutralizing potency of HeB02 sera against its self-virus was 2^12^, while the neutralizing potency against SD19/11103 was 2^3^, indicating a low cross-reactivity between SD19/11103 and HeB02, and an obvious antigenic difference. In conclusion, neutralization experiments showed a significant difference in antigenicity between SD19/11103 and HeB02 (Table 1).

### 3.5. SD19/11103 Induced Severe Lesions in Immunized Chickens

**Figure 5:**
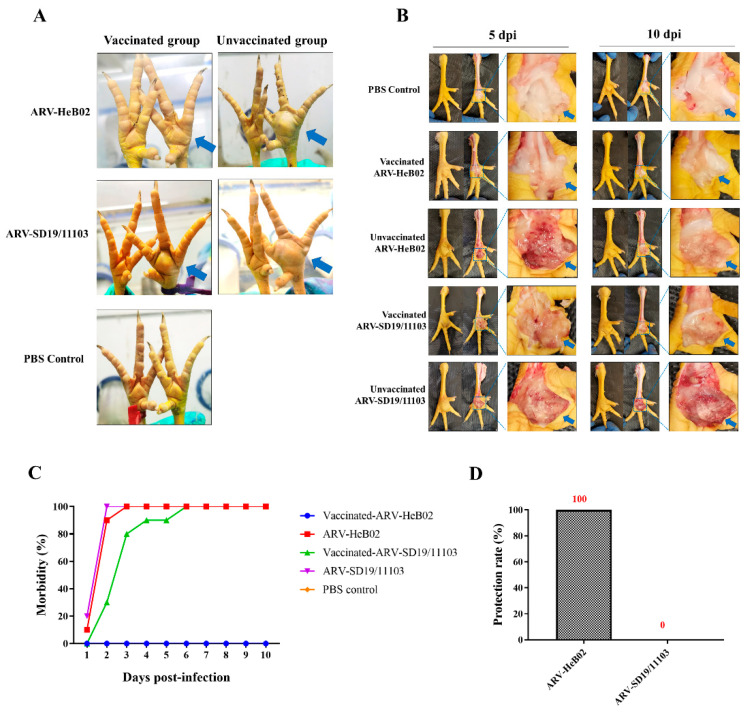
The immunoprotective effect of ARV commercial vaccine against novel variant strain SD19/11103 was evaluated using 3-week-old SPF chicks. (**A**) The gross lesions of footpads of different groups were observed after challenged with SD19/11103 or HeB02. After challenge with SD19/11103, SPF chickens in both vaccinated and unvaccinated groups showed obvious gross lesions. The vaccinated and HeB02-challenged group showed intact footpads and no obvious visual lesions. To clearly show the gross lesions, the swelling footpads have been indicated with blue arrows. (**B**) The pathological changes of footpads were observed after dissection at 5 dpi and 10 dpi. After challenge with SD19/11103, SPF chickens in both vaccinated and unvaccinated groups showed gelatinous exudates and obvious congestion in the footpads at 5 dpi and 10 dpi. No obvious pathological changes were observed after challenge with HeB02 in the vaccinated group. To clearly show the pathological changes of footpads, parts of the images have been enlarged and indicated with blue arrows. (**C**) Morbidity of SPF chickens after challenged with the SD19/11103 or HeB02 strain. (**D**) The protection rate of SPF chickens after challenged with the SD19/11103 or HeB02 strain.

To evaluate the immune-protective effect of a commercial vaccine against the novel ARV variant strain, SPF chickens vaccinated with the commercial vaccine were challenged with the SD19/11103 or HeB02 strain. Compared with the blank control group, the vaccinated and HeB02-challenged group (group 2) showed intact footpads and no obvious visual lesions, whereas the vaccinated and SD19/11103-challenged group (group 1) showed significant footpad swelling and lesions (Figure 5A). After challenge with SD19/11103, SPF chickens in both vaccinated and unvaccinated groups showed obvious gross lesions. After dissection, these two groups showed gelatinous exudates and obvious congestion in the footpads at 5 dpi and 10 dpi (Figure 5B). In the vaccinated group, no obvious pathological changes were observed after challenge with HeB02, but the unvaccinated group (group 4) showed similar symptoms to the SD19/11103-challenged group. These results indicated that the commercial ARV vaccine could not provide complete protection against SD19/11103.

## 4. Discussion

ARV infection is associated with diseases such as viral arthritis, tenosynovitis, and malabsorption syndrome [17]. ARVs cause direct economic losses owing to their associated morbidity and mortality [7]. In contrast, immunodeficiency in surviving chickens increases the risk of secondary infection with other viruses, bacteria, and parasites, thereby exacerbating damage [18]. 

Protection against disease in chickens has historically been achieved through vaccination with a combination of commercial live and inactivated vaccines. The primary objectives for vaccination are to prevent vertical transmission, provide maternal derived antibodies to progeny and prevent clinical disease. In China, this disease has been successfully controlled in the past few years with the use of vaccines. However, since 2013, ARV infection has been increasingly detected in broilers in China [7]. The severe immunosuppression caused by the ARV variants poses a new threat to the poultry industry. The emergence of ARV variants has led to a continuous increase in the incidence of ARV infections, which has seriously affected the production performance of commercial chickens, such as weight gain and feed–meat ratio, and is also the reason for vaccination failure [19], threatening the development of the poultry industry. 

In this study, a variant strain was isolated from the swollen joint tissues of broilers from a poultry in Shandong Province, China, where all broilers were immunized with the ARV vaccines. RT-PCR and IFA results confirmed the successful isolation of the virus (ARV-SD19/11103). To evaluate the pathogenicity of ARV variants, 1-day-old chickens were infected with the SD19/11103 and HeB02. The SD19/11103 strain showed obvious pathogenicity to chickens. However, compared to the HeB02 strain, the death time of the SD19/11103 variant strain was delayed, joint swelling symptoms were milder, and the mortality rate was lower, indicating the difference in pathogenicity between the variant strain and the genotype cluster I ARV strain.

Protein σC is encoded by the third open reading frame of the ARV S1 genome fragment [20]. The σC protein is a major determinant of virulence and antigenic variation and is critical for analyzing strains as it is the most variable protein in ARV and is considered the only viral protein with type-specific neutralizing immunity [4,14,21,22,23,24]. Changes in σC gene/protein homology may lead to changes in ARV virulence [25,26,27]. Therefore, our molecular characterization focused on the σC gene sequences. Genetic evolutionary analysis of the σC gene showed that SD19/11103 was located in the genotype cluster I, but not in the same sub-cluster as the S1133 vaccine strain. It is worth noting that the genetic distance between SD19/11103 and genotype cluster I strains (such as S1133, 2408, 1733, or HeB02) is large, and the amino acid sequences homology of the σC gene among them was 73.1–76.1%. The cross-protection effect is weakened when the amino acid difference between the virus strain and the vaccine strain is ≥5% [28]. This means that the available commercially genotype cluster I vaccines may no longer provide good protection against the SD19/11103 variant strain. To better understand the molecular information related to antigenic variation and virulence of the novel variant of ARV, we cloned and sequenced the genome of the SD19/11103 strain. Phylogenetic analysis further confirmed that SD19/11103 was a new ARV variant. Furthermore, SD19/11103 exhibited 33 amino acid differences in the σC gene compared to genotype cluster I ARV strains (including the S1133 vaccine strain). It has been reported that residues 210–246 of the ARV σC protein are involved in apoptosis induction through interactions [25,26,27,29], and these conserved mutations may affect the role of σC in inducing apoptosis. Changes in σC receptor-binding and apoptosis-inducing ability may help change its virulence; however, further research is needed to investigate the effects of these specific amino acid mutations on its function. 

Antigenic variations and mismatches are the main factors leading to immune failure. There are antigenic differences between the novel ARV variant and the genotype cluster I strains. To verify this, the antigenic relationship between the novel ARV variant and the genotype cluster I strain was evaluated using a serum cross-neutralization assay. The results revealed a clear difference in antigenicity between SD19/11103 and HeB02. This novel ARV variant may evade the immune protection provided by commercial ARV vaccines. To test this hypothesis, animal experiments were conducted. These results were consistent with our hypothesis that current commercial vaccines could not provide effective protection against variant strains. The paw pads of the immunized group were significantly swollen after challenge with the variant strain, whereas the immunized group challenged with the genotype cluster I strain showed no clinical symptoms of the disease, and the immune protection rate was 100%. The above experimental results are consistent with the results of the cross-neutralization test, which once again proves that there is antigenicity difference between the variant strain and genotype cluster I strain. This may be the reason for the circulation of ARV disease in vaccinated chickens.

The immune failure associated with traditional vaccination may pose significant challenges and complexities in effectively preventing and controlling of ARV infections. Based on local representative viruses, vaccine production for each phylogenetic group may be more effective [30].

## 5. Conclusions

This study confirmed the serious pathogenic effects of a novel ARV variant on vaccinated chickens from both natural and laboratory perspectives. The commercially available vaccines are unable to provide adequate levels of protection against emerging variants, which may be the main cause of ARV variants circulating in China. Our study further characterized the antigenic differences between the novel ARV variant and genotype cluster I ARV strain. Our findings explain ARV infections in immunized flocks and highlight the need to develop new vaccines against this novel ARV variant.

## Figures and Tables

**Table 1:** Results of cross-neutralization assays.

Sera	Virus	
	ARV-HeB02	ARV-SD19/11103
ARV-HeB02	2^12^	2^3^
ARV-SD19/11103	-	2^6^

## Data Availability

All the data are presented in this study.

## Supplementary Materials

The following supporting information can be downloaded at: https://www.mdpi.com/article/10.3390/v15091800/s1, Table S1: Reference ARV strains and the accession numbers in GenBank; Table S2: Characteristic of amino acid differences in σC protein of ARV genotype cluster Ⅰ strains and SD19/11103. The asterisk indicates the same amino acid residue as the novel ARV variant SD19/11103.
