# Supplementary material for: A Novel Variant of Avian Reovirus Is Pathogenic to Vaccinated Chickens
Source: Viruses. 2023 Aug 24;15(9):1800. doi: 10.3390/v15091800 (PMC10538029; doi:10.3390/v15091800)
Supplement: Supplementary file 1 [file viruses-15-01800-s001.zip › Supplementary Table S1.pdf]

The Supplementary Table S1 Reference ARV strains and the accession numbers in GenBank.

No	Name of strains	Genbank accession no.	Origin	Cluster	No	Name of strains	Genbank accession no.	Origin	Cluster
1	S1133	L39002	USA	Ⅰ	20	ISR5215	FJ793531	Israel	Ⅰ
2	1733	AF004857	USA	Ⅰ	21	ISR5225	FJ793546	Israel	Ⅰ
3	JR1	EF122836	USA	Ⅰ	22	916	AF297214	China	Ⅱ
4	2408	AF204945	China	Ⅰ	23	GEL13a98M	AF354226	Netherlands	Ⅱ
5	601SI	AF204947	China	Ⅰ	24	ISR528	FJ793523	Israel	Ⅱ
6	GuangxiR1	KC183744	China	Ⅰ	25	TARV-MN3	KF872234	USA	Ⅱ
7	GuangxiR2	KF741732	China	Ⅰ	26	TARV-Crestview	KF872238	USA	Ⅱ
8	GX-2010-1	KJ476705	China	Ⅰ	27	TARV-O'NEIL	KF872231	USA	Ⅱ
9	GX110058	KF741742	China	Ⅰ	28	GEL13b98M	AF354227	Netherlands	Ⅲ
10	GX110116	KF741752	China	Ⅰ	29	ISR525	FJ793539	Israel	Ⅲ
11	HB10-1	KP288833	China	Ⅰ	30	ISR5233	FJ793549	Israel	Ⅲ
12	JS01	KX451230	China	Ⅰ	31	42563-4-2005	DQ872801	USA	Ⅲ
13	MS01	KY860636	China	Ⅰ	32	AVS-B	FR694197	Hungary	Ⅳ
14	SD09-1	KP288853	China	Ⅰ	33	K1600657	MK583337	USA	Ⅳ
15	SD10-1	KP288863	China	Ⅰ	34	918	AF297215	China	Ⅴ
16	C-98	EF057397	China	Ⅰ	35	1017-1	AF297216	China	Ⅴ
17	T-98	EF057398	China	Ⅰ	36	03200-12	KP727785	USA	Ⅵ
18	HeB02	KX451231	China	Ⅰ	37	03476-12	KP727784	USA	Ⅵ
19	GEL12 98M	AF354225	Netherlands	Ⅰ	38	19981-13	KR856993	USA	Ⅵ
