# Supplementary material for: A Novel Variant of Avian Reovirus Is Pathogenic to Vaccinated Chickens
Source: Viruses. 2023 Aug 24;15(9):1800. doi: 10.3390/v15091800 (PMC10538029; doi:10.3390/v15091800)
Supplement: Supplementary file 1 [file viruses-15-01800-s001.zip › Supplementary Table S2.pdf]

The Supplementary Table S2. Characteristic of amino acid differences in σC protein of ARV genotype cluster I strains and SD19/11103. The asterisk indicates the same amino acid residue as the novel ARV variant SD19/11103.

Strain	Cluster	σC																																
		26	27	46	49	51	54	60	68	70	77	78	83	86	94	95	98	102	111	112	126	137	147	156	203	236	243	245	256	261	264	285	291	310
SD19/11103	I	N	P	S	Q	V	M	D	T	R	V	T	S	I	T	T	A	E	S	G	S	A	N	T	V	G	A	S	M	S	I	S	V	N
GEL12 98M (AF354225)	I	*	*	*	*	*	*	*	*	*	*	*	*	*	*	*	*	*	*	*	*	*	*	*	*	*	*	*	*	*	*	*	*	*
ISR5225 (FJ793546)	I	*	*	*	*	*	*	*	*	*	*	*	*	*	*	*	*	*	*	*	*	*	*	*	*	*	*	*	*	*	*	*	*	*
ISR5215 (FJ793531)	I	*	*	*	*	*	*	*	*	*	*	*	*	*	*	*	*	*	*	*	*	*	*	*	*	*	*	*	*	*	*	*	*	*
HeB02 (KX451231)	I	S	H	L	R	I	I	N	M	H	A	N	R	V	S	I	T	D	A	A	T	S	D	A	M	N	V	L	I	A	V	A	A	D
S1133 (L39002)	I	S	H	L	R	I	I	N	M	H	A	N	R	V	S	I	T	D	A	A	T	S	D	A	M	N	V	L	I	A	V	A	A	D
1733 (AF004857)	I	S	H	L	R	I	I	N	M	H	A	N	R	V	S	I	T	D	A	A	T	S	D	A	M	N	V	L	I	A	V	A	A	D
2408 (AF204945)	I	S	H	L	R	I	I	N	M	H	A	N	R	V	S	I	T	D	A	A	T	S	D	A	M	N	V	L	I	A	V	A	A	D
601SI (AF204947)	I	S	H	L	R	I	I	N	M	H	A	N	R	V	S	I	T	D	A	A	T	S	D	A	M	N	V	L	I	A	V	A	A	D
JR1 (EF122836)	I	S	H	L	R	I	I	N	M	H	A	N	R	V	S	I	T	D	A	A	T	S	D	A	M	N	V	L	I	A	V	A	A	D
HB10-1 (KP288833)	I	S	H	L	R	I	I	N	M	H	A	N	R	V	S	I	T	D	A	A	T	S	D	A	M	N	V	L	I	A	V	A	A	D
JS01 (KX451230)	I	S	H	L	R	I	I	N	M	H	A	N	R	V	S	I	T	D	A	A	T	S	D	A	M	N	V	L	I	A	V	A	A	D
MS01 (KY860636)	I	S	H	L	R	I	I	N	M	H	A	N	R	V	S	I	T	D	A	A	T	S	D	A	M	N	V	L	I	A	V	A	A	D
SD09-1 (KP288853)	I	S	H	L	R	I	I	N	M	H	A	N	R	V	S	I	T	D	A	A	T	S	D	A	M	N	V	L	I	A	V	A	A	D
GX-2010-1 (KJ476705)	I	S	H	L	R	I	I	N	M	H	A	N	R	V	S	I	T	D	A	A	T	S	D	A	M	N	V	L	I	A	V	A	A	D
SD10-1 (KP288863)	I	S	H	L	R	I	I	N	M	H	A	N	R	V	S	I	T	D	A	A	T	S	D	A	M	N	V	L	I	A	V	A	A	D
C-98 (EF057397)	I	S	H	L	R	I	I	N	M	H	A	N	R	V	S	I	T	D	A	A	T	S	D	A	M	N	V	L	I	A	V	A	A	D
GuangxiR1 (KC183744)	I	S	H	L	R	I	I	N	M	H	A	N	R	V	S	I	T	D	A	A	T	S	D	A	M	N	V	L	I	A	V	A	A	D
GuangxiR2 (KF741732)	I	S	H	L	R	I	I	N	M	H	A	N	R	V	S	I	T	D	A	A	T	S	D	A	M	N	V	L	I	A	V	A	A	D
GX110058 (KF741742)	I	S	H	L	R	I	I	N	M	H	A	N	R	V	S	I	T	D	A	A	T	S	D	A	M	N	V	L	I	A	V	A	A	D
T-98 (EF057398)	I	S	H	L	R	I	I	N	M	H	A	N	R	V	S	I	T	D	A	A	T	S	D	A	M	N	V	L	I	A	V	A	A	D
GX110116 (KF741752)	I	S	H	L	R	I	I	N	M	H	A	N	R	V	S	I	T	D	A	A	T	S	D	A	M	N	V	L	I	A	V	A	A	D
